# Mutational spectra and mutational signatures: Insights into cancer aetiology and mechanisms of DNA damage and repair

**DOI:** 10.1016/j.dnarep.2018.08.003

**Published:** 2018-11

**Authors:** David H. Phillips

**Affiliations:** a MRC-PHE Centre for Environment and Health, King's College London, UK; b NIHR Health Protection Research Unit in Health Impact of Environmental Hazards at King’s College London in Partnership with Public Health England, Department of Analytical, Environmental and Forensic Sciences, School of Public Health and Environmental Sciences, Faculty of Life Sciences and Medicine, King’s College London, UK

**Keywords:** 1,2DCP, 1,2dichloropropane, DCM, dichloromethane, AAN, aristolochic acid nephropathy, BEN, Balkan endemic nephropathy, TC-NER, transcription coupled nucleotide excision repair, iPS, induced pluripotent stem, ROS, reactive oxygen species, Mutagen, Mutational signature, Whole genome sequencing

## Abstract

Reporter gene assays, in which a single mutation from each experiment can contribute to the assembly of a mutation spectrum for an agent, have provided the basis for understanding the mutational processes induced by mutagenic agents and for providing clues to the origins of mutations in human tumours. More recently exome and whole genome sequencing of human tumours has revealed distinct patterns of mutation that could provide additional clues for the causative origins of cancer. This can be tested by examining the mutational signatures induced in experimental systems by putative cancer-causing agents. Such signatures are now being generated *in vitro* in a number of different mutagen-exposed cellular systems. Results reveal that mutagens induce characteristic mutation signatures that, in some cases, match signatures found in human tumours. Proof of principle has been established with mutational signatures generated by simulated sunlight and aristolochic acid, which match those signatures found in human melanomas and urothelial cancers, respectively. In an analysis of somatic mutations in cancers for which tobacco smoking confers an elevated risk, it was found that smoking is associated with increased mutation burdens of multiple different mutational signatures, which contribute to different extents in different tissues. One of these signatures, mainly found in tissues directly exposed to tobacco smoke, is attributable to misreplication of DNA damage caused by tobacco carcinogens. Others likely reflect indirect activation of DNA editing by APOBEC cytidine deaminases and of an endogenous clock-like mutational process. The results are consistent with the proposition that smoking increases cancer risk by increasing the somatic mutation load although direct evidence for this mechanism is lacking in some cancer types. Thus, next generation sequencing of exomes or whole genomes is providing new insights into processes underlying the causes of human cancer.

## 1 Introduction

Cancers arise as a result of somatic mutations. All tumours have been found to contain multiple mutations, including point mutations, insertions, deletions and gene rearrangements. Mutations that occur in critical genes involved in processes that maintain the integrity of cells (the ‘driver’ mutations) and that when mutated confer a growth advantage, can be considered causative in transforming a normal cell into a malignant one. At the same time, many other mutations are present in tumours (the ‘passenger’ mutations), accumulated as a result of genomic instability induced by the transformed or malignant state, or as bystander mutations arising from the same influences that gave rise to the ‘driver’ mutations, but occurring in regions of the genome that do not cause the phenotypic changes causing the emergence of the transformed state. Tumours are clonal, having arisen from a single cell that has acquired abnormal growth characteristics as a result of mutation, but can nevertheless display heterogeneity due to later acquisition of more mutations. A cancer genome can be considered to contain the history of mutagenic processes that have occurred throughout the life of the cancer patient, both before and after the acquisition of a neoplastic transformation phenotype by the progenitor cell of the tumour.

## 2 Mutational spectra – multiple studies in single genes

Experimental studies have, until recently, focused on the analysis of mutations in a single gene, whereby mutated cells were selected on the basis of a growth advantage under the conditions of the assay, or by producing progeny with a readily identified marker, such as a cellular dye.

Some of these systems arise out of short-term tests to identify mutagens, *i.e.* those that simply identify whether or not a test compound or agent is mutagenic. However, further investigation to identify the nature of the mutations that have arisen forms the basis of generating a mutation spectrum, by the acquisition of mutations from many experiments, often one at a time. These include bacterial studies based on the Ames test, identifying mutations in the *his* operon of *Salmonella typhimurium* [1]. Such analyses have been applied to both single chemicals and complex mixtures [2]. A mutagen will produce the same type of substitution mutation in all organisms, which reflects the conservation of DNA repair and replication processes through evolution [3].

An early eukaryotic system for mutagenicity testing focused on the *HPRT* gene, whose mutation in response to mutagens was measured in mouse, hamster and human cells lines; cells in which *HPRT* mutations are rendered resistant to 6-thioguanine and will form colonies when cultured in media containing it. Detection of the mutations requires PCR amplification of the gene and direct sequencing of the transcripts [4].

The use of transgenic rodent mutation assays has been a useful and informative way of determining mutational spectra *in vivo* [5]. Mutations are detected by the introduction of the *lacZ* (Muta^™^mouse) or *lacI* gene (Big Blue® mouse or rat) into animals, which is then recovered from genomic DNA of treated rodents and packaged into a *λ* phage vector and infected into *E. coli.* Mutant colonies are then identified by means of a colour change from wild-type colonies; for mutagen testing purposes the mutation frequency is sufficient; however sequencing of the *lacZ, lacI* or the smaller *cII* gene in these mutant plaques provides the data to enable a mutation spectrum to be compiled. Another transgenic rodent model, *gpt* delta mouse and rat, has been developed that allows detection of point mutations by virtue of resistance to 6-thioguanine toxicity [5].

None of the genes mentioned thus far have anything to do with carcinogenesis. Indeed, in some of the systems, the reporter gene is not even expressed (*e.g. lacZ* and *lacI*), although paradoxically this can be an advantage in that the mutations detected have not undergone any selective pressure.

Attention has thus turned to considering mutations in ‘driver’ genes. Although mutational spectra based on *Kras* and *Hras* proto-oncogenes will be biased because only some types of mutations at certain codons can result in an activated oncogene, nevertheless different agents induce mouse tumours with distinctly different patterns of mutations in these genes, which can be informative about mechanism of carcinogenesis and origins of tumours [6]. Differences in mutational spectra in premalignant papillomas and metastatic skin tumours induced in mice by 7,12-dimethylbenz[*a*]anthracene have shed light on the clonal evolution of metastasis in this experimental model [7].

More complete spectra can be obtained from analysis of the tumour suppressor gene *TP53*, which carries mutations in more than 50% of human tumours [8]. Nearly 30,000 *TP53* mutations in human tumours are catalogued in a database (http://p53.iarc.fr). From this compendium some distinct patterns have emerged pointing to specific causative agents: UV light in the case of skin cancers [9,10], aflatoxin B1 in the case of hepatocellular carcinoma [11,12], benzo[*a*]pyrene and other polycyclic aromatic hydrocarbons in the case of smokers’ lung cancer [13] and aristolochic acid (a plant chemical) in the case of urothelial cancer (described in detail later in this article).

The generation of a mouse line with exons 4–9 of the human *TP53* gene (the Hupki mouse) [14] led to the development of an assay to detect mutations in sequences of the human gene in clones of immortalised mouse embryo fibroblasts. Using this approach agents such as UV light induced C to T and CC to TT mutations, benzo[*a*]pyrene (a tobacco carcinogen) induced G to T transversions, and aristolochic acid induced A to T transversions. In each case these mutations and their distribution (spectrum) in the *TP53* gene sequence correlated with those seen in human tumours where these agents were suspected of being causative [15].

Impressive though these associations are, the number of human carcinogens identified by scrutinising one gene only, either experimentally or in human tumours, is limited to just these few cases. Generating mutation spectra by the accumulation of mutations one by one from each experiment or tumour is not an efficient process. Given that it is now known that many tumours contains hundreds to thousands of mutations, the question arises as to what information can be gained, and what patterns can emerge, from an examination of all of them?

## 3 Mutational signatures – multiple mutations across the exome or whole genome

### 3.1 Mutational processes in human cancer

**Fig. 1:**
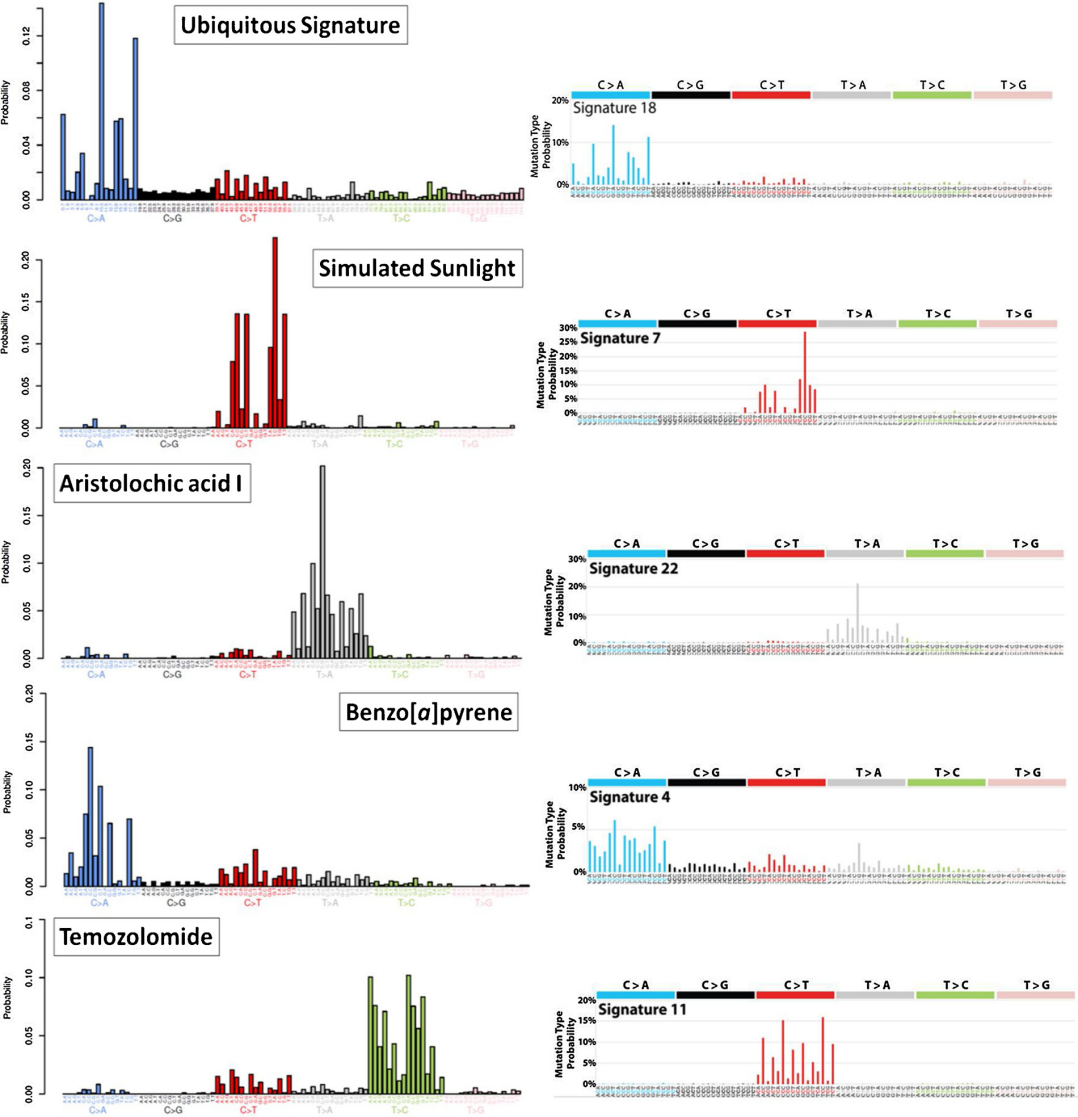
Mutational signatures generated experimentally and extracted from human tumours by whole genome sequencing. Each signature has 96 components, comprised of six possible base pair substitutions, each of which has four possible 5′ neighbouring bases and four possible 3′ neighbouring bases. The experimental signatures, shown on the left, were produced from induced human pluripotent stem cells exposure to mutagens [31]. The human tumour signatures, on the right, are from the COSMIC database (http://cancer.sanger.ac.uk/cosmic/signatures). The figure shows that propagation of cells in culture without treatment generates a signature similar to the COSMIC signature 18; simulated sunlight produces a signature similar to COSMIC signature 7; aristolochic acid produces a signature similar to COSMIC signature 22; benzo[*a*]pyrene produces a signature similar to COSMIC signature 4 (the “smoking” signature); temozolomide produces a signature that is distinctly different from COSMIC signature 11.

From sequence analysis of over 7000 human cancers (mostly by exome sequencing, but with around 7% by whole genome sequencing) Alexandrov et al. [16] extracted 22 distinct mutational signatures, a signature being a representation of the 96 possible substitution mutations possible within at a base pair in the middle of a trinucleotide (*i.e.* there are six possible point mutations, four possible bases 5′ to it and another four 3′ to it, making 6 × 4 × 4 = 96 possible events) (see Fig. 1). When the analysis was expanded to ∼12,000 human tumours, the number of signatures increased to 30 (http://cancer.sanger.ac.uk/cosmic/signatures). Imminently, with a further increase in the database to include more than 23,000 tumours of 71 cancer types, containing more than 83 million mutations, around 49 substitution signatures, together with 17 indel signatures and 11 dinucleotide (tandem) mutation signatures, will be reported (L. Alexandrov, personal communication).

Already, examination of these signatures, in association with the aetiology, where known, of the cancers from which they originate, has revealed a great amount about the mutagenic processes involved in carcinogenic processes. Around a third of the 30 signatures have been attributed to endogenous mutagenic processes, such as the activity of the APOBEC family of deaminases and deamination of 5-methylcytosine, deficiencies in DNA repair processes, including mismatch repair and homologous recombination, or defective DNA polymerases [17]. A further six are attributed to exposure to mutagenic agents, including tobacco carcinogens, UV radiation, aflatoxin B1, alkylating chemotherapy drugs and the plant carcinogen aristolochic acid; these are discussed in more detail below. The causes of the remaining 14 signatures are not yet identified. With the identification of additional signatures, as mentioned above, it still remains the case that approximately half of them are of unknown cause or origin (L. Alexandrov, personal communication).

Identifying the processes underlying the uncharacterised signatures and/or determining the agents that cause them will shed new light on the causes of cancer. In pursuit of this, a number of approaches have been used to generate mutagen-induced signatures *in vitro*. These have been useful in providing experimental evidence to validate or, in some cases question, the causative mechanisms assigned to some of the signatures in human tumours. What follows are examples of these approaches.

### 3.2 Aflatoxin mutational signature

In the COSMIC catalogue, signature 24 has been found only in liver cancer and is associated with reported exposure to aflatoxin B1, a known cause of hepatocellular carcinoma. The strong transcriptional bias in the mutations, which are predominantly GC-TA transversions, also supports the hypothesis that these are the result of misreplication of aflatoxin-DNA adducts, which would be subject to transcription-coupled nucleotide excision repair (TC-NER). In order to test the hypothesis, mutations induced by aflatoxin B1 in two different human cells lines, in liver tumours in wild-type mice and also in mice with a hepatitis B surface antigen transgene, were investigated by whole genome sequencing [18]. HepaRG and HepG2 cells that had been chronically exposed to aflatoxin B1 and cloned had predominantly GC-TA substitutions, with a strong bias for the G being on the non-transcribed strand. The bias decreased from the 5′ to 3′ ends of transcripts, in line with evidence that the effectiveness of TC-NER decreases thus [19]. The most enriched sequences were in both cases TGC, TGG and AGC, the same as found in the signatures, consistent with COSMIC signature 24, of newly sequenced hepatocellular carcinomas from Qidong, China, where exposure to aflatoxin B1 is well documented. There were similarities with the mouse liver tumours, but also greater diversity in the mutational landscapes, likely evidence of additional processes, such as the involvement of the hepatitis B surface antigen in the mouse strain engineered to express it [18]. Taken together, these findings greatly strengthen the causative association between aflatoxin B1 exposure and liver cancer.

### 3.3 Haloalkane exposure in print workers

Following a report of a high incidence of cholangiocarcinoma among print workers in Japan, whole exome sequencing was carried out on a number of cases [20]. The mutational signatures of each of four printing workers’ cholangiocarcinomas were dominated by CG-TA transversions with a substantial strand bias and they all had a prominent trinucleotide signature mutation GCY to GTY (where Y is a pyrimidine, C or T), followed by NCY to NTY and NAY (where N is any base) that were seen only to a minor extent in control cases of cholangiocarcinoma. Two of the solvents to which the workers were exposed, 1,2-dichloropropane (1,2-DCP) and dichloromethane (DCM), were mutagenic to *S. typhimurium* TA100, with CG-TA transversions accounting for 58–71% of the 1,2-DCP-induced mutations and 47% of those induced by DCM. The predominant trinucleotide mutation sequence for 1,2-DCP was NCC to NTC, *i.e.* overlapping with the 2nd most common pattern in the printing workers, whereas the signature for DCM did not have anything in common with the tumour signatures. It can be concluded from these studies that the mutational signatures in the four print workers display common features that are indicative of exposure to a chemical that forms bulky adducts that are recognised by the nucleotide excision repair pathway (hence the mutational strand bias); among candidate mutagens, 1,2-DCP gives a mutational signature in bacteria that partially recapitulates the signature in the tumours. The discrepancies between the two systems point to more complex underlying processes that have yet to be elucidated, and possibly to additional mutagens that have yet to be identified [20]. The authors attempted to generate a mutational signature for 1,2-DCP in human epithelial cell lines, but were unsuccessful; thus the lack of a suitable mammalian system, thus far, in which to generate mutational signatures from 1,2-DCP also places limitations on the conclusions to be drawn from this study.

### 3.4 Mutational signatures of tobacco smoking

Tobacco smoke contains at least 60 different carcinogens and smoking is causally linked to 17 different cancers. In an analysis of several thousand cancer genome sequences from both smokers and non-smokers, it was found that the smokers had significantly higher numbers of base substitutions than the non-smokers for all the 17 cancer types together and for several individual cancer types, namely larynx, liver, kidney and lung adenocarcinoma [21]. In tumours of tissue directly exposed to tobacco smoke (*e.g.* lung and larynx) the COSMIC signature 4 was prominent. As noted earlier, this signature is similar to that produced by benzo[*a*]pyrene in cells *in vitro* and this, coupled with the strong strand bias indicative for TC-NER, suggests that the signature arises from the misreplication of DNA damage (adducts) formed by carcinogens present in tobacco smoke. Several other signatures were found to be present at elevated levels of mutation in the smokers’ tumours relative to the non-smokers, especially signatures 2, 5, 13 and 16.

Signatures 2 and 13, which feature GC-AT and GC-CG mutations, respectively, at TpC dinucleotides, are considered to be the result of overactivity of APOBEC deaminases in DNA editing. The reason for this overactivity, which is found in many tumour types, is not clear but may be the result of retrotransposon movement, cellular response to foreign DNA and local inflammation [22]. The frequency of mutations of signature 5, which is found in all tumour types and has a predominance of AT-GC (with transcriptional bias) and also GC-AT mutations, correlates with age of diagnosis in non-smokers [23]. The increase in its frequency in smokers’ lung squamous and adenocarcinomas, larynx, pharynx, oral cavity, oesophageal squamous, bladder, liver and kidney cancers suggests that the “clocklike” process that generates it is accelerated by smoking. Signature 16 is associated mainly with liver cancer and its origin is unknown. It is characterised by AT-GC mutations at ApT dinucleotides, which exhibit a strong transcriptional strand bias.

The numbers of mutations that accumulate per cell annually per pack year of smoking in various tissues was calculated to be: for lung, 150; for larynx, 97; for pharynx, 39; for oral cavity, 23; for bladder, 18; for liver, 6. Interestingly, this order correlates with the ranking of odds ratios for the risk of smoking-related cancer in these organs. The study demonstrates that tobacco smoking enhances, or accelerates, endogenous mutagenic processes in some susceptible tissues, but not others, and it also induces mutations directly by carcinogens present in tobacco smoke, although this mechanism appears to be confined mainly to tissues that are directly exposed to tobacco smoke [21]. The findings reveal the complexity of the mechanisms of tobacco carcinogenesis, which can vary between different tumour sites.

### 3.5 The aristolochic acid mutational signature

Aristolochic acid is a natural compound found in many plants that are used as traditional herbal remedies and health supplements. Although it was known to be carcinogenic to animals, it was not until it was accidently included in a slimming regimen at high doses in Belgium in the 1990s, with the consequence that many of those treated succumbed to renal failure and urothelial cancer, that the cancer risk to humans was appreciated. Within a short time it was noted that the pathology of aristolochic acid nephropathy (AAN), as it became known, was similar to that of Balkan endemic nephropathy (BEN), a hitherto idiopathic condition occurring in many rural regions of Southeast Europe [24], but where human exposure to aristolochic acid now appears to result from contamination of cereal crops by *Aristolochia clematitis* growing wild in the regions [25].

Evidence that aristolochic acid is the causative agent of both AAN and BEN came first from investigation of mutation spectra and subsequently from mutational signatures. In tumours associated with both the human diseases, *TP53* mutations were frequently AT-TA transversions, a mutation that is rare in urothelial cancers in general. In experiment systems aristolochic acid forms DNA adducts predominantly with adenine residues in DNA, leading to AT-TA transversions [24,26]. Comparison of the sites in *TP53* at which AT-TA transversions occurred in mouse embryo fibroblasts and in tumours from BEN patients showed a significant similarity [27].

Progressing from this single gene analysis, it has now been demonstrated conclusively from whole exome and whole genome sequencing of urothelial tumours that aristolochic acid gives rise to a distinctive and diagnostic mutational signature (COSMIC signature 22) that is dominated by AT-TA transversions [28,29]. The evidence that the tumours have been caused by aristolochic acid-induced mutations is further supported by the detection of DNA adducts on aristolochic acid in the tumour and tumour-adjacent tissue, and the experimental evidence that aristolochic acid produces the same signature in mouse embryo fibroblasts [30] and human induced pluripotent stems cells [31].

There is now evidence that aristolochic acid exposure in the Balkans may not be confined to the predominantly rural areas where BEN occurs. Whole genome sequencing of 14 Romanian renal cell carcinoma cases from non-BEN areas revealed that they all exhibited mutational signatures consistent with aristolochic acid exposure, a result also supported by the presence of aristolochic acid-DNA adducts in non-tumour renal tissue [32].

AAN is now recognised as a worldwide health issue. More recently, evidence has appeared for the involvement of aristolochic acid in cancer at additional sites. Analysis of bladder tumours from Asia found that the aristolochic acid signature was present in a number of them, including 2 out of 2 from Taiwan, 1 out of 11 from Singapore and 3 out of 99 from China [33]. Exome analysis of hepatocellular carcinomas revealed the presence of signature 22 in 78% of those from Taiwan, 49% of those from China and in 29% of those from elsewhere in Southeast Asia [34]. In addition, the signature was also detected in 13% of cases from Korea and 2.7% of those from Japan.

It should be noted that the AT-TA component of the mutational signature that aristolochic acid induces is not unique. A very similar signature is produced *in vitro* by a polycyclic aromatic hydrocarbon, dibenzo[*a,l*]pyrene [31]. However, in the case of aristolochic acid, the AT-TA mutations account for >85% of the mutations detected, whereas for dibenzo[*a,l*]pyrene it accounts for only 50%. Nevertheless, this sounds a note of caution that overreliance on evidence from mutational signatures, in the absence of evidence of exposure, could lead to misclassification of the cause(s) of cancer cases.

### 3.6 A compendium of experimentally induced mutational signatures

In order to examine systematically the mutational patterns associated with treatment of a comprehensive selection of environmental mutagens generated under highly controlled conditions, 79 chemicals/agents were selected on the basis that they are environmental or therapeutic agents that have been classified by the International Agency for Research on Cancer (IARC) as human carcinogens (Group 1), probable human carcinogens (Group 2 A) or possible human carcinogens (Group 2B) [31]. Human induced pluripotent stem (iPS) cells were then exposed to each of them, clonally expanded and subjected to whole genome sequencing. In all cases, including untreated controls, the mutational signatures contained a component that was very similar to COSMIC signature 18 (Fig. 1), which has been attributed to mutagenesis by reactive oxygen species (ROS) in combination with base excision repair defects from *MUTYH* germline mutations [35,36]. After subtraction of the background signature from the pooled mutations for each treatment, the remaining mutations of approximately half of the agents tested also gave rise to distinct mutational signatures. Three of these, produced by simulated sunlight, aristolochic acid and benzo[*a*]pyrene, correlated closely with signatures 7, 22 and 4, respectively, reproducing what had previously been observed with mouse cells (Fig. 1) [30].

In all, some 40 new signatures have been generated from mutagenic agents [31]. Some of the agents gave similar signatures, as might be expected from their known patterns of DNA adduct formation. For example, the *N*-ethyl-*N*-nitrosourea, *N*-methyl-*N*-nitrosourea and temozolomide signatures were markedly similar to each other; however they were markedly different form COSMIC signature 11 (Fig. 1), which has been attributed to temozolomide therapy. The reason for this difference is not yet clear, but it may be that there are DNA repair processes that are more prominent in stem cells than in somatic cells, or *vice versa*. Another case of similar compounds giving similar signatures was the platinum complexes cisplatin and carboplatin, whose DNA adducts are identical to each other. However there are also examples for members of different classes giving rise to quite similar signatures, for example benzo[*a*]pyrene (a polycyclic aromatic hydrocarbon) and 2-amino-1-methyl-6-phenylimidazo[4,5-*b*]pyridine (PhIP, a heterocyclic amine). Compounds of the same chemical class could also give rise to significantly different signatures, such as the polycyclic aromatic hydrocarbons, ostensibly due to differences in the proportions of adducts formed at guanine and adenine residues in DNA. Nevertheless there was great similarity between the signatures of each of three polycyclic aromatic hydrocarbons and their respective bay-region diol-epoxides, providing evidence that the latter are indeed the major intermediates in the metabolic activation of PAHs in human cells [31].

The agents that give rise to bulky DNA adducts, subject to TC-NER, nearly all showed significant strand bias in their mutational signatures. An exception is simulated sunlight, which did not display any strand bias, even though COSMIC signature 7, which it matches, does have the strand bias expected of TC-NER of UV-induced DNA lesions. A similar lack of strand bias was found in the UV-induced signature, also highly similar to signature 7, in mouse cells [30]. The reason for this anomaly is not clear at present.

## 4 Concluding remarks

The rapidly expanding database of mutational landscapes in human tumours is providing significant new information on the molecular events underlying the hallmarks of cancer. Until recently reporter gene assays, in which a single mutation (usually) from each experiment can contribute to the assembly of a mutation spectrum for an agent, have provided a basis of our understanding of mutational processes in human tumours. However we have now entered an era in which technologies for whole genome sequencing have advanced to the point where it is economically feasible to observe all the mutations produced in such assays, not just those in the reporter gene, and also to go further by generating mutational signatures in clones generated in systems without a selection pressure [30,37,38]. A number of different approaches are already evolving using, for example, cloned chicken cells [39], human induced pluripotent stem cells [31], and *C. elegans* [40] and showing the potential to reveal the role of exogenous and endogenous electrophiles, and or DNA repair and DNA damage response processes, in carcinogenesis.

With this massive increase in data acquisition and in statistical power coupled with the wealth of data that can be obtained from whole genome sequencing of human tumours, it is anticipated that mutational signatures produced experimentally will provide new insights into the agents and processes underlying the causes of human cancer.

## Conflict of interest

The author has no conflict of interest.
